# Help or hindrance? The evolutionary impact of whole‐genome duplication on immunogenetic diversity and parasite load

**DOI:** 10.1002/ece3.6987

**Published:** 2020-11-22

**Authors:** Ellen A. Bell, Jo Cable, Claudio Oliveira, David S. Richardson, Levi Yant, Martin I. Taylor

**Affiliations:** ^1^ School of Biological Sciences University of East Anglia Norwich UK; ^2^ School of Biosciences Cardiff University Cardiff UK; ^3^ Departmento de Morfologia Instituto de Biosiências/UNESP São Paulo Brazil; ^4^ Department of Cell and Developmental Biology John Innes Centre Norwich UK; ^5^ Present address: Future Food Beacon of Excellence and the School of Life Sciences University of Nottingham Nottingham UK

**Keywords:** immunogenetic diversity, infectious disease, sympatric communities, toll‐like receptors, whole‐genome duplication

## Abstract

Whole‐genome duplication (WGD) events occur in all kingdoms and have been hypothesized to promote adaptability. WGDs identified in the early history of vertebrates, teleosts, and angiosperms have been linked to the large‐scale diversification of these lineages. However, the mechanics and full outcomes of WGD regarding potential evolutionary impacts remain a topic of debate. The Corydoradinae are a diverse subfamily of Neotropical catfishes with over 170 species described and a history of WGDs. They are divided into nine mtDNA lineages, with species coexisting in sympatric—and often mimetic—communities containing representatives of two or more of the nine lineages. Given their similar life histories, coexisting species of *Corydoras* might be exposed to similar parasite loads and because of their different histories of WGD and genome size they provide a powerful system for investigating the impacts of WGD on immune diversity and function in an animal system. Here, we compared parasite counts and the diversity of the immune‐related toll‐like receptors (TLR) in two coexisting species of *Corydoras* catfish (*C. maculifer* and *C. araguaiaensis*), one diploid and one putative tetraploid. In the putative tetraploid *C. araguaiaensis*, we found significantly lower numbers of parasites and significantly higher diversity (measured by both synonymous and nonsynonymous SNP counts) in two TLR genes than in the diploid *C. maculifer*. These results provide insight into how WGD may impact evolution, in this case by providing greater immunogenetic diversity.

## Graphical abstract

The full evolutionary impacts of whole‐genome duplication (WGD) events on species (especially in animal systems) remain unclear. In our study, we look at parasite abundance and immunogenetic diversity in two species of *Corydoras* catfish, a diploid (Corydoras maculifer, right) and a putative (Corydoras araguaiaensis, left) allotetraploid that coexist in a sympatric community. We found the putative tetraploid species exhibited fewer external parasites and had significantly higher immune gene diversity.

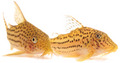

## 1 INTRODUCTION

Whole‐genome duplication (WGD) events have been identified across the animal and plant kingdoms: There have been as many as four ancient WGD events in the angiosperms, with up to 70% of plants having a history of polyploidy (Masterson, 1994; van de Peer et al., 2009). Vertebrates have undergone two rounds of WGD during their evolutionary history, with an additional event detected in the teleost lineage, termed the fish/teleost specific genome duplication (Dehal & Boore, 2005; Santini et al., 2009). Multiple additional lineage‐specific WGD events have occurred in a number of fish and amphibians, including salmonids and *Xenopus* species (Mable et al., 2011). WGD events have been implicated in major evolutionary transitions, such as those seen in flowering plants around the K‐T boundary (van de Peer et al., 2009), and the increased potential for tolerance or robustness in a changing environment (van de Peer et al., 2017).

While the occurrence of both ancient and recent WGDs across kingdoms is well accepted, the importance of WGDs in adaptation is unclear. Opposing schools of thought posit that WGD events have led to increased diversification rates and biological novelty or, alternatively, have negligible evolutionary impact (Baduel et al., 2018; Furlong & Holland, 2004; Ohno, 1970; Stebbins, 1940; van de Peer et al., 2009). In vitro evolutionary experiments in yeast have shown that WGD can significantly increase the rate of adaptation as a result of more beneficial mutations (Selmecki et al., 2015). Outside of laboratory conditions, WGD or smaller scale duplication events are increasingly being associated with neofunctionalization of duplicated genes (where duplicate genes assume a new role). Examples of this include the roles of the *scn4aa* gene, which codes for Na^+^ channel α‐subunits and has been co‐opted in electric organs of mormyroid and gymnotiform fish, and that of the DEF‐like gene family, which has been associated with unique flower formation of orchids (Moriyama & Koshiba‐Takeuchi, 2018).

Whole‐genome duplication events may also confer more immediate impacts if greater immune gene diversity in polyploids leads to increased resistance to pathogens (King et al., 2012). Genes that encode immune proteins are frequently highly polymorphic (even in diploids), and this high diversity can favor pathogen‐mediated balancing selection (Netea et al., 2012; Phillips et al., 2018; Spurgin & Richardson, 2010). Duplicated immune genes may lead to redundancy, reduce purifying selection on novel mutations in duplicated copies and thus potentially allow recognition of a wider range of pathogens, increased expression of immunogenic proteins and/or an increase in immunogenic novelty.

One multigene group of immune genes is the toll‐like receptors (TLRs) which belong to the group of type I transmembrane proteins and recognize extracellular pathogen‐associated molecular patterns (PAMPs), initiating downstream immune mechanisms (Zhao et al., 2013). Genes encoding TLRs tend to be highly polymorphic and genome‐wide association studies (GWAS) have detected links between specific polymorphisms in TLRs and disease susceptibility (Netea et al., 2012; Skevaki et al., 2015). Furthermore, major functional specializations in TLRs have been identified as following ancient gene duplication events prior to the divergence of protostomes and deuterostomes (Hughes & Piontkivska, 2008).

The Corydoradinae are a species‐rich subfamily of Neotropical catfishes (Family Callichthyidae), split into nine distinct mtDNA lineages, which exhibit highly variable genome sizes, with C‐values ranging from 0.5 to ~4 pg (Alexandrou et al., 2011; Oliveira et al., 1992). A recent study using restriction site associate DNA (RAD) markers found evidence for multiple WGD events across the nine lineages along with differences in haplotype retention, transposable element (TE) abundance and single nucleotide polymorphism (SNP) read ratios (Marburger et al., 2018). This study identified two potential WGD events, one at the base of mtDNA lineage 2 (encompassing lineages 2–9) and a second more recent event at the base of mtDNA lineage 9 (Marburger et al., 2018). A unique aspect of these catfish is their propensity to live in mixed sympatric communities of up to three species, often from different lineages and with varying genome sizes and histories of WGD (Alexandrou et al., 2011). This makes them a powerful model for examining the impact of WGD in an animal system. *Corydoras maculifer* (mtDNA lineage 1; diploid) and *C. araguaiaensis* (mtDNA lineage 9; putative tetraploid) coexist in the same small streams in the Rio Araguaia region of Brazil (Alexandrou et al., 2011). Therefore, these species share the same environment, have similar life histories, and occupy similar trophic levels. Accordingly, they are likely exposed to the same pathogens.

Here, we utilize this mixed ploidy community to test for relationships between ploidy, parasite load, and immune gene diversity—specifically TLR diversity. We found a significantly greater parasitic load in the diploid species and significantly greater immunogenetic diversity in the putative tetraploid species. These findings suggest either greater resistance in the tetraploid species or greater tolerance in the diploid.

## 2 MATERIALS AND METHODS

### 2.1 Sampling, dissection, and parasite counts

Individuals of *Corydoras maculifer* (haploid C value = 0.65 pg, diploid (Alexandrou, 2011)) and *C. araguaiaensis* (haploid C value = 4.36 pg, putative tetraploid, (Alexandrou, 2011)) were collected from the wild in the Rio das Mortes drainage of the Araguaia River in Mato Grosso state, Brazil in 2012 and 2015. Fish were euthanized by anesthetic overdose (benzocaine at a concentration of 100 mg/L) and preserved individually in 100% ethanol (Alexandrou et al., 2011). Samples from 2015 were dissected in the field; stomach, liver, and digestive tract were preserved separately in 100% ethanol with the aim of genetically identifying any parasites, but for logistic reasons, this was not possible within the scope of this study. As a result, samples underwent significant handling in the field and therefore ectoparasites could not be accurately assessed, and we acknowledge that the parasite counts for individual fish will be conservative estimates. Other preserved samples (from 2012) were then added to the 2015 cohort. Samples from both years (*C. maculifer*, *n* = 20 and *C. araguaiaensis,* *n* = 41) were later dissected and tissues (external, body cavity, liver, stomach, and digestive tract) assessed for macroparasites using a dissecting microscope with fiber optic illumination. A preliminary survey of gill tissue, from a subset of samples (*n* = 7 *C. maculifer*, *n* = 14 *C. araguaiaensis*), was assessed for parasites; however, none were detected so assessment of this tissue was curtailed. Parasites were removed, identified to phylum, counted and stored separately in 100% ethanol. For each host species, the proportion of fish infected and number of parasites were recorded and then analyzed. Differences between the proportions of infected host species were tested using Fisher's exact tests implemented in R (R version 3.4.1).

To control for host size and partially control for host age (assuming a correlative relationship between size and age; Hordyk et al., 2015; Mahé et al., 2016), counts for all parasite taxa, host species, and host standard length (the distance between tip of snout and base of tail) were used to fit a Poisson generalized linear model (GLM) to model the number of parasites per mm of host using the GLM function in R (R version 3.4.1). Number of parasites was measured as a function of host species, and host length was included in the model as an offset. The model was refitted using a quasi‐Poisson distribution to allow for the dispersion parameter to be estimated from the data. Parasites were categorized as either external microsporidian cysts or endoparasites, and the GLM was fitted to data from both of these categories and to total sum parasite counts. Parameter estimates from these GLMs were then used to predict parasite abundance per millimeter of host species for each of the parasite categories and plotted using ggplot2 (version 2.2.1).

### 2.2 Immune gene sequencing

DNA was extracted from fin clips from *C. maculifer* (*n* = 17) and *C. araguaiaensis* (*n* = 36) using a modification of the salt extraction protocol after Aljanabi and Martinez (1997) and Sunnucks and Hales (1996). Four sets of forward and reverse primers were designed to amplify c. 2.5 kb fragments of both TLR1 and TLR2 in *C. maculifer* and *C. araguaiaensis* based on transcriptome sequences (Taylor et al., unpublished, primer details given in Table S1). PCR amplifications were composed of 1.25 μl of 10 μmol of each forward and reverse primer, 12.5 μl of PCR Master Mix (Phusion high fidelity PCR Master Mix), and 2 μl of extracted DNA made up to a final solution volume of 25 μl with dH_2_O. PCR conditions were as follows: initial denaturation of 98°C for 30 s, and then a secondary denaturation of 98°C for 10 s, primer‐specific annealing temperatures (see Table S1) for 30 s, extension at 72°C for 120 s for 35 cycles and a final extension step at 72°C for 5 min. Sequencing libraries were produced following a modification of Peterson et al. (2012). Sequencing was performed on an Illumina NextSeq platform.

Sequencing data for TLRs 1 and 2 were quality checked, de‐multiplexed first by ligation index then by PCR barcode and cleaned using Cutadapt (v 1.13; Martin, 2011) and Trimmomatic (v. 0.2.36, Bolger et al., 2014) using default settings for both. Reads from one individual of each species were mapped (using BWA‐mem with default settings, v.0.7.12; Li & Durbin, 2009) to the TLR genes in a draft *C. maculifer* genome (Taylor et al., unpublished) to produce reference TLR assemblies for each species. Raw reads were then mapped back to the relevant species TLR assembled reference genes using BWA‐mem using the default parameters. Variants were called with FreeBayes on default settings but requiring a minimum of 5 observations per SNP (v. 1.1.0; Garrison & Marth, 2012) prior to filtering using FreeBayes CSV. Reference genes were put in to open reading frames, and variants were subdivided into synonymous and nonsynonymous SNP categories. Data were plotted using ggplot2 (version 2.2.1) in R studio (R version 3.4.1). Wilcoxon tests were used to investigate whether there was a difference in synonymous and nonsynonymous SNP counts within each TLR and between *C. maculifer* and *C. araguaiaensis* and also between TLR1 and TLR2 within study species.

Haplotype counts were estimated per species using two methods; firstly, short‐range phasing (long‐range phasing being unfeasible with these data due to the history of WGD in one of the species) and secondly; read‐depth ratio. QualitySNPng (Nijveen et al., 2013) was used to phase short segments of both TLRs and to estimate minimum haplotypes for each individual. Secondly, the frequency of read‐depth ratios per SNP for each TLR across individuals was calculated, following the expectation that diploids have average reference to alternative SNP ratios of 0.5, triploids 0.33 and 0.66, and tetraploids 0.25, 0.5, and 0.75 based on the dosage of alleles in different ploidies, following the methods of Yoshida et al. (2013).

## 3 Results

### 3.1 Parasite burden

A total of 18/20 (80%) of the *Corydoras maculifer* and 27/41 (66%) of the *C. araguaiaensis* harbored parasites. Parasites were found in all tissue types examined (body cavity, stomach, lower digestive tract, and liver). The only ectoparasite found was a single isopod on a specimen of *C. araguaiaiensis,* which though noted here, was removed from most downstream analysis and only noted in total parasite counts, because handling issues noted in the methods, are likely to have removed most ectoparasites in the field. External surface microsporidian cysts were significantly more common in *C. maculifer* (8/20, 40%) compared to *C. araguaiaensis* (1/41, 5%) (Fisher's exact test, *n* = 61, *p* < .0003, Figure S1). For infected hosts, the total number of parasites was also generally higher in *C. maculifer* but none of the comparisons were statistically significant (Figure S2).

**FIGURE 1:**
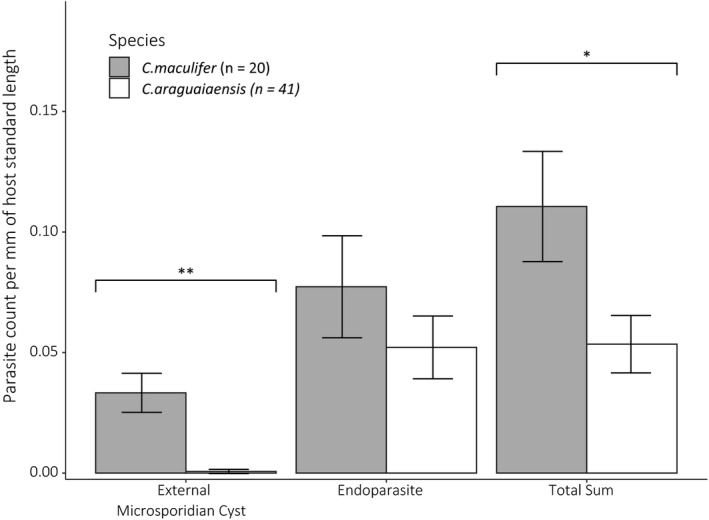
Parasite count measured as a function of host species (*Corydoras maculifer* and *C. araguaiaensis*) and with host length included as an offset in the GLMs, error bars represent standard error. Parameter estimates from GLMs were used to predict parasite counts per millimeter of host (calculated from standard length, from the tip of the snout to the base of the tail) and plotted. Count data were over dispersed so the GLM was refitted using a quasi‐Poisson distribution (**p < .004, *p < .02)

When accounting for body size, parasite counts were significantly higher in *C. maculifer* than in *C. araguaiaensis* for external surface microsporidan cysts and total number of parasites (Figure 1, GLM, effect of host species designation, estimated Std = −3.91, Error = 1.31, *t* value = −2.98, *p* < .004 and estimated Std = −0.72, Error = 0.30, *t* value = −2.39, *p* < .02 for external surface microsporidan cysts and total parasite abundances, respectively).

### 3.2 TLR variation

**FIGURE 2:**
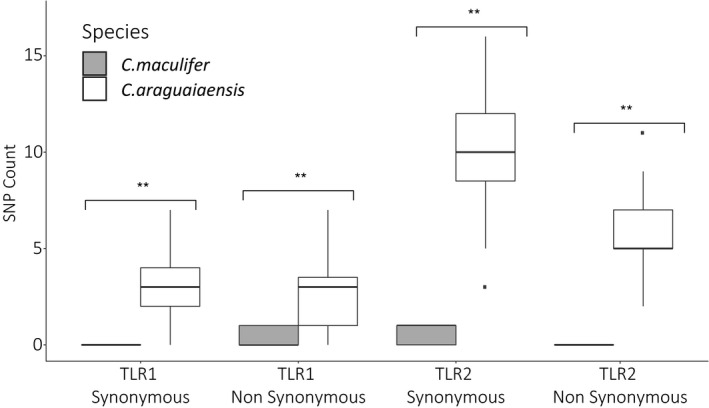
SNP counts in the samples of *Corydoras maculifer* (*n* = 17) and *C. araguaiaensis* (*n* = 35) for two TLR genes (TLR1 and TLR2). SNPs are divided by substitution type (i.e., synonymous and nonsynonymous) with Wilcoxon tests (**p < .01)

Sequencing of TLR1 and TLR2 across both species produced a total of 876,570 single reads (GC content = 46%). Following clean‐up and demultiplexing, mapped read counts were >500 reads per locus per individual and mean depth was 62.3X (*SD* 28) in *C. maculifer* and 103.5X (*SD* 23) in *C. araguaiaensis* across each of the 2.5 kb fragments. Variant calling in TLR1 and TLR2 detected one SNP in *C. maculifer* in each TLR gene. In *C. araguaiaensis*, 42 SNPS were identified in TLR1 and 114 SNPs in TLR2 (Figure 2). At both TLR1 and TLR2, *C. araguaiaensis* had a significantly higher number of both synonymous (TLR1, Wilcoxon test, *W* = 34, *p* < .01, TLR2, Wilcoxon test, *W* = 0, *p* < .01) and nonsynonymous SNPS (TLR1, Wilcoxon test, *W* = 70, *p* < .01, TLR2, Wilcoxon test, *W* = 0, *p* < .01) than *C. maculifer*. Overall, TLR2 had significantly more SNPs then TLR1 (Wilcoxon Test: *W* = 16.5, *p* < .01) in *C. araguaiaensis* with no difference in *C. maculifer* (no test performed on 1 SNP in each TLR).

**FIGURE 3:**
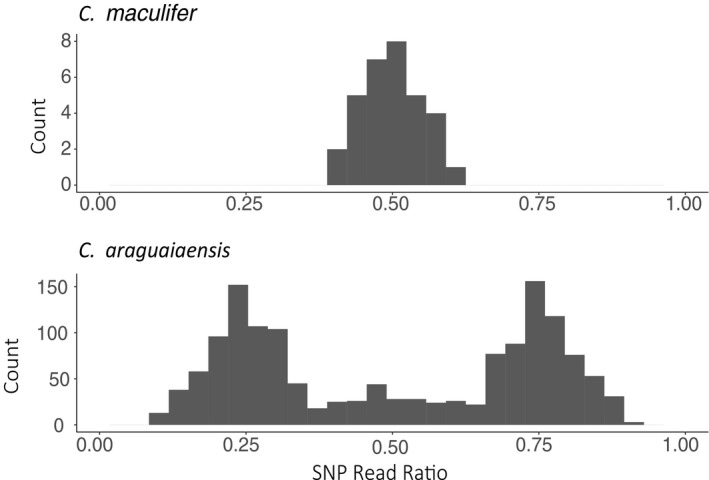
SNP read ratios (the proportion of reference and alternative reads at each SNP site) at two TLR loci (TLR1 and TLR2) averaged across a population of *Corydoras maculifer* (*n* = 17) and another of C. araguaiaensis (n = 35)

**FIGURE 4:**
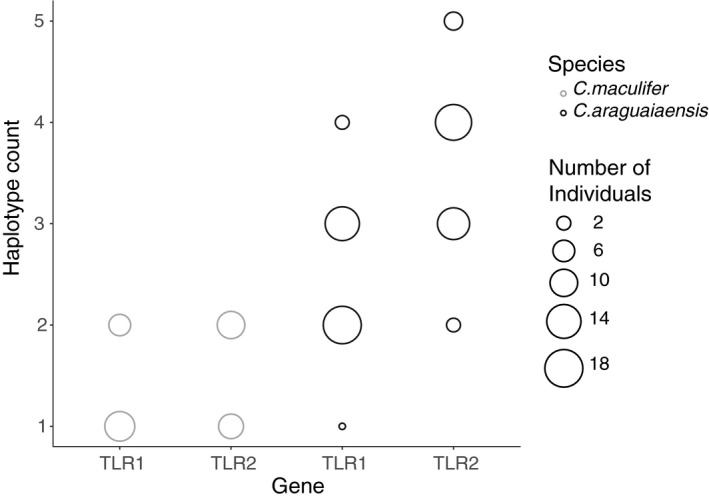
Minimum inferred haplotype counts at TLR1 and TLR2 loci in *Corydoras maculifer* (*n* = 17) and *C. araguaiaensis* (*n* = 35) as derived by short‐range phasing by QualitySNPng and verified manually

SNP read ratios showed a clear peak at 0.5 in *C. maculifer*, which is the expected average for diploid individuals, and peaks close to 0.25 and 0.75 in *C. araguaiaensis* as predicted for tetraploid individuals (Figure 3). Short‐range phasing of SNPs into haplotypes indicated that *C. araguaiaensis* (putative tetraploid) had retained four different copies of both TLR1 and TLR2 with as many as 5 TLR2 haplotypes in 4 individuals. A maximum of two haplotypes were found in *C. maculifer* (Figure 4). Positional distribution of SNPs across the TLR gene fragments showed no clear patterns (for synonymous or nonsynonymous SNPs) in *C. araguaiaensis*. Furthermore, the majority of SNPs had low frequencies in *C. araguaiaensis* (Figures S3 and S4), but each individual had its own unique combination of SNPs across all haplotypes.

## 4 DISCUSSION

Here, we compared parasite counts and immune gene diversity in two sympatrically coexisting species of *Corydoras* catfishes with differing genome sizes and histories of WGD. The proportion of hosts infected did not differ significantly between host species unless considering just external surface microsporidan cysts; then, a significantly greater proportion of *C. maculifer* (diploid) carried an infection. When host size was accounted for, parasite counts were significantly higher for both external surface microsporidan cysts and for total parasite counts in *C. maculifer* (diploid), than in *C. araguaiaensis* (putative tetraploid). In line with our expectation based on WGD, higher genetic diversity was identified at both TLR1 and TLR2 in *C. araguaiaensis* than in *C. maculifer*. Therefore, a significant difference in immune gene diversity and associated parasite resistance and/or tolerance was identified between two host species with different WGD histories but which share the same environment.

The number of parasites harbored by an organism is determined by many intrinsic and extrinsic factors. Intrinsic factors can be divided into host resistance, an organism's capacity to limit parasite burden, and host tolerance, the capacity of an organism to limit damage caused by infection (Råberg et al., 2009). The patterns observed in our study indicate greater resistance in *C. araguaiaensis* or greater tolerance in *C. maculifer*, although without experimental approaches, these two alternatives cannot be distinguished.

Immunogenetic data showed almost no variation in the TLRs in *C. maculifer* (diploid), with similar levels of variation to those observed in bottlenecked and/or threatened populations (Gilroy et al., 2017; Grueber et al., 2015). Conversely, significantly higher numbers of synonymous and nonsynonymous SNPs were observed in *C. araguaiaensis* (putative tetraploid). In addition, when comparing complete SNP profiles among individuals across the population, no two individual *C. araguaiaensis* were identical. This indicates that no individuals shared all four haplotypes at either of the two TLRs in the sample investigated here. This high among individual diversity could be the result of heterozygote advantage and negative frequency dependence (King et al., 2012), or the result of excess haplotypes masking mutations and genetic drift therefore reducing the effectiveness of selection and purging (Comai, 2005; Otto & Whitton, 2000).

Short‐range phasing and SNP ratios confirmed that *C. maculifer* harbored a maximum of two haplotypes per individual and suggested that *C. araguaiaensis* generally had four, with a maximum of five, haplotypes per individual. While different types of duplication event (whole genome, tandem, proximal, retrotransposed, DNA transposed, and dispersed) have been associated with expansion of gene families there are subtle differences in their evolutionary signatures (Qiao et al., 2018; Wang et al., 2011). The results of these analyses were in agreement with Marburger et al., 2018 which identified peaks at 0.25, 0.5, and 0.75 from in analysis of SNP frequency distribution across the *C. araguaiaensis* genome. This suggests tetraploidy and supports the theory that additional haplotypes in *C. araguaiaensis* originated from WGD (Marburger et al., 2018). These analyses confirmed that, despite the normal trend for rapid rediploidization via gene fractionation following WGD (Soltis et al., 2015), additional haplotypes of TLR1 and TLR2 have been maintained in *C. araguaiaensis* without genomic evidence of silencing or degradation (except for one SNP causing a stop codon in three individuals). The additional fifth haplotype (in 4 individuals) was unexpected and could be the result of tandem duplication or relicts from more ancient WGD events.

Population‐wide polymorphism levels were generally low in both *Corydoras* species, with no clear pattern regarding their location along the TLR gene. Other population‐wide studies have found signatures of positive or balancing selection in TLRs and have linked them to the need for variation in PAMP binding regions (Gilroy et al., 2017). The signatures described in the current study are more suggestive of cumulative drift being preserved in haplotypes that are surplus to requirement rather than expansion and neofunctionalization of TLR genes.

In summary, significantly lower levels of parasite abundance and significantly higher levels of diversity, in the form of both synonymous and nonsynonymous SNPs, in two Toll‐like receptor (TLR) genes were found in a putative tetraploid catfish species compared to a cohabiting diploid species. Although it is not possible, within the confines of this study, to distinguish between increased resistance of the polyploid species or increased tolerance of the diploid this study provides further insight into evolutionary implications of WGD in relation to immunogenetic diversity and pathogen response.

## CONFLICT OF INTEREST

We declare no conflicts of interest.

## AUTHOR CONTRIBUTION

**Ellen Alicia Bell:** Conceptualization (equal); Data curation (lead); Formal analysis (lead); Investigation (lead); Methodology (lead); Project administration (equal); Visualization (lead); Writing‐original draft (lead); Writing‐review & editing (lead). **Jo Cable:** Data curation (supporting); Formal analysis (supporting); Investigation (supporting); Methodology (supporting); Writing‐review & editing (supporting). **Claudio Oliveira:** Resources (supporting); Writing‐review & editing (supporting). **David Richardson:** Conceptualization (supporting); Investigation (supporting); Project administration (supporting); Resources (supporting); Supervision (supporting); Writing‐review & editing (supporting). **Levi Yant:** Formal analysis (supporting); Investigation (supporting); Methodology (supporting); Resources (supporting); Writing‐review & editing (supporting). **Martin Taylor:** Conceptualization (equal); Formal analysis (supporting); Funding acquisition (lead); Investigation (supporting); Methodology (supporting); Project administration (equal); Resources (equal); Supervision (lead); Writing‐original draft (supporting); Writing‐review & editing (supporting).

### OPEN RESEARCH BADGES

This article has been awarded Open Data and Open Materials Badges. All materials and data are publicly accessible via the Open Science Framework at Parasite count and prevalence data along with accompanying scripts for GLMs and variant calls for TLR1 and TLR2 sequence data have been deposited in Dryad: https://doi.org/10.5061/dryad.gf1vhhmmb. The sequence data for TLR1 and TLR2 across all samples has been deposited in the National Center for Biotechnology Information (NCBI) Sequence Read Archive (SRA): BioProject 641559.

## Supporting information

Fig S1

Fig S2

Fig S3

Fig S4

Table S1

## Data Availability

Parasite count and prevalence data along with accompanying scripts for GLMs and variant calls for TLR1 and TLR2 sequence data have been deposited in Dryad: https://doi.org/10.5061/dryad.gf1vhhmmb. The sequence data for TLR1 and TLR2 across all samples have been deposited in the National Center for Biotechnology Information (NCBI) Sequence Read Archive (SRA): BioProject 641559.
