# Supplementary figures and images for: Help or hindrance? The evolutionary impact of whole‐genome duplication on immunogenetic diversity and parasite load
Source: Ecol Evol. 2020 Nov 22;10(24):13949–56. doi: 10.1002/ece3.6987 (PMC7771170; doi:10.1002/ece3.6987)

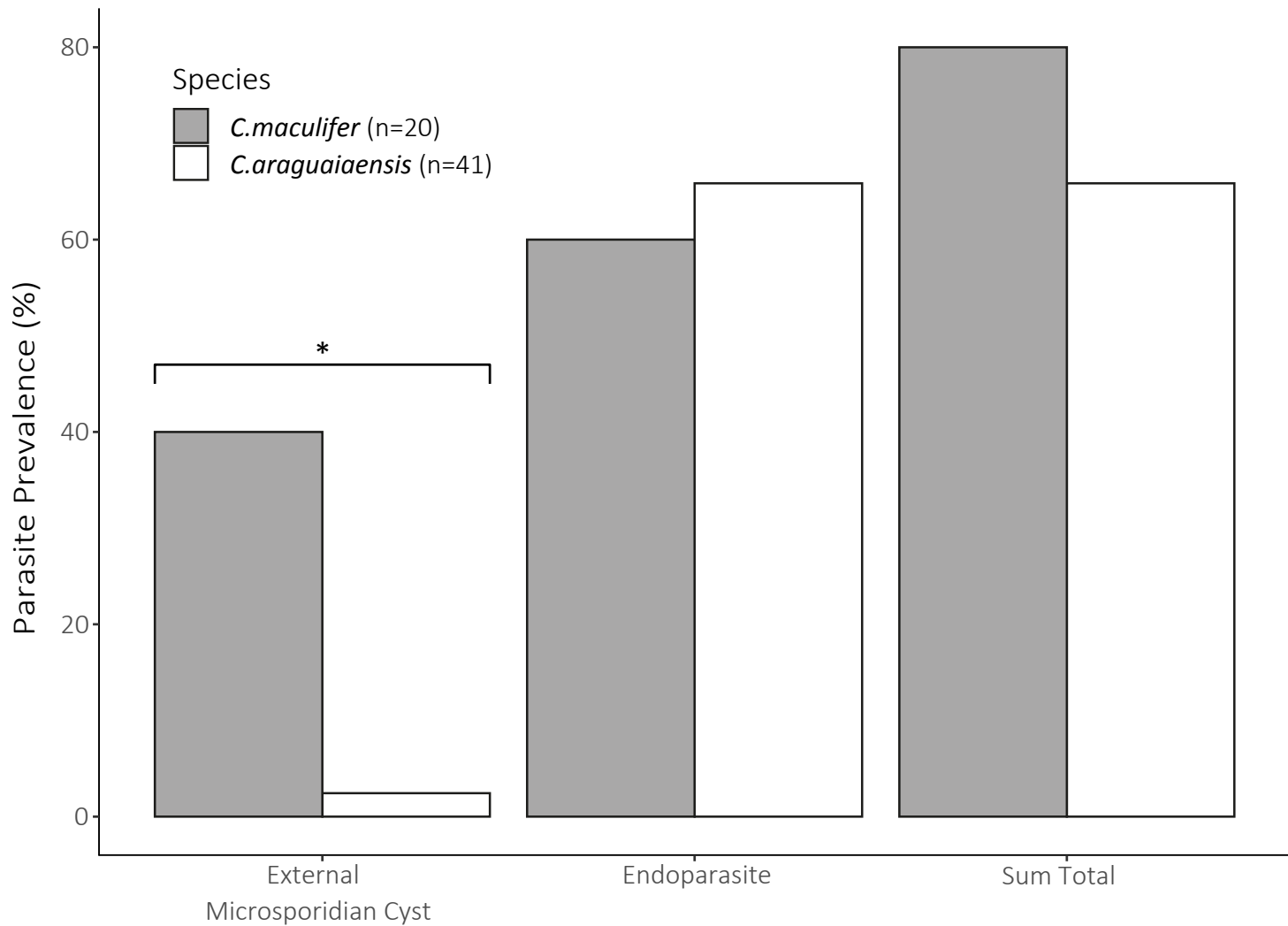

Supplement: Supplementary file 1 — Fig S1 [file ECE3-10-13949-s001.pdf]

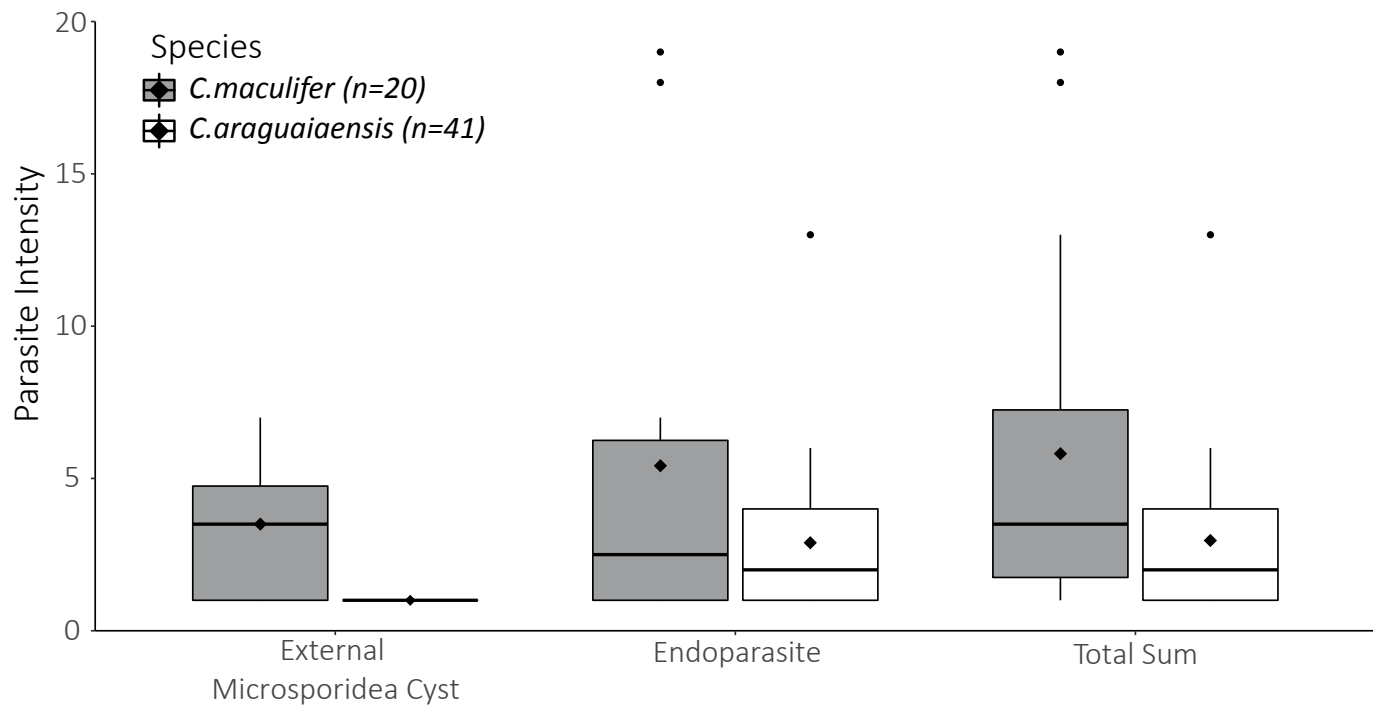

Supplement: Supplementary file 2 — Fig S2 [file ECE3-10-13949-s002.pdf]
