# Supplementary material for: Help or hindrance? The evolutionary impact of whole‐genome duplication on immunogenetic diversity and parasite load
Source: Ecol Evol. 2020 Nov 22;10(24):13949–56. doi: 10.1002/ece3.6987 (PMC7771170; doi:10.1002/ece3.6987)
Supplement: Supplementary file 3 — Fig S3 [file ECE3-10-13949-s003.pdf]

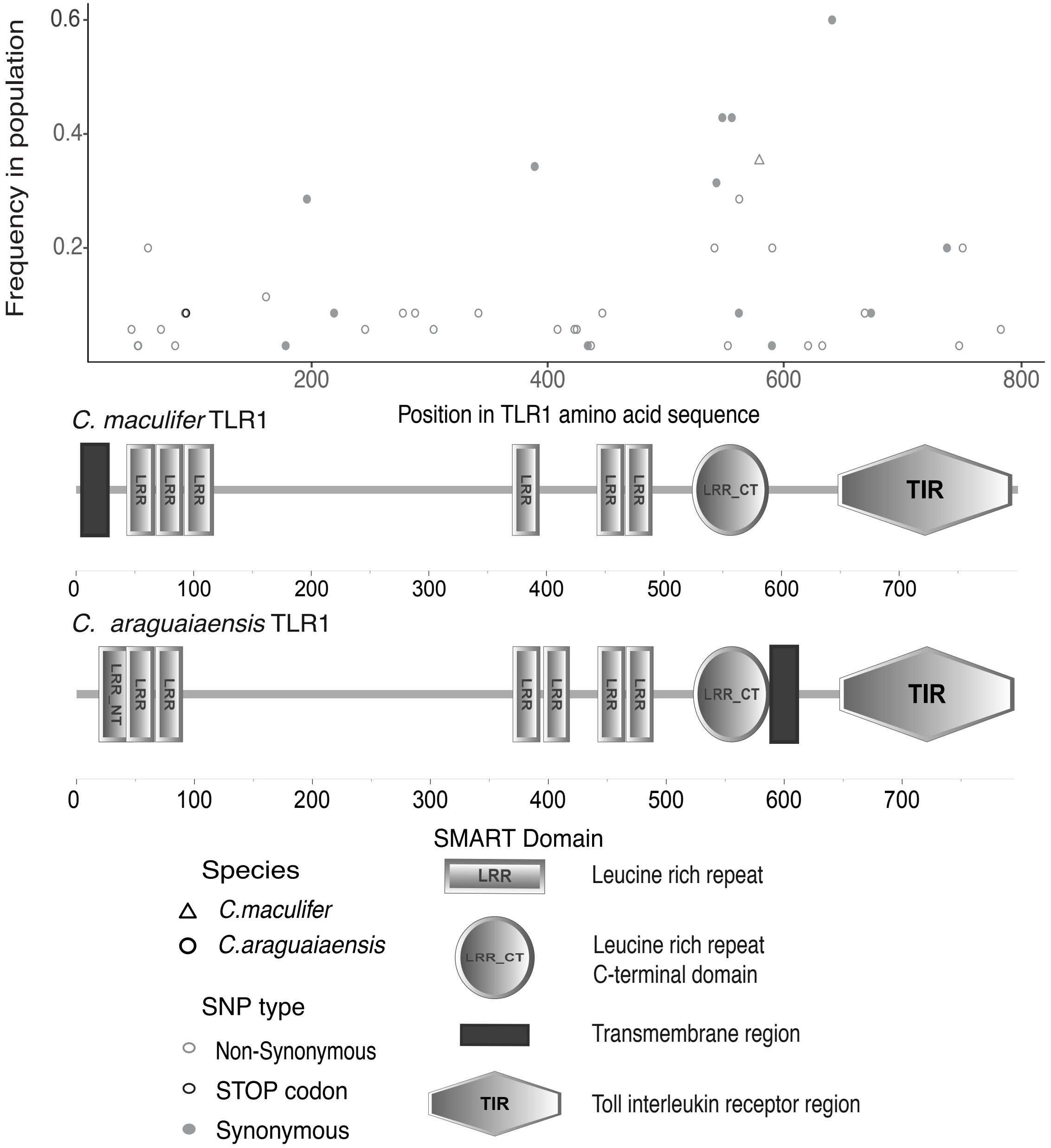

Frequency in population
0.6
0.4
0.2
200
400
600
800
Position in TLR1 amino acid sequence
C. maculifer TLR1
LRR
LRR_CT
TIR
0
100
200
300
400
500
600
700
C. araguaiaensis TLR1
LRR_NT
Species
C.maculifer
C.araguaiaensis
SNP type
Non-Synonymous
STOP codon
Synonymous
SMART Domain
Leucine rich repeat
Leucine rich repeat C-terminal domain
Transmembrane region
Toll interleukin receptor region
