# Supplementary material for: Help or hindrance? The evolutionary impact of whole‐genome duplication on immunogenetic diversity and parasite load
Source: Ecol Evol. 2020 Nov 22;10(24):13949–56. doi: 10.1002/ece3.6987 (PMC7771170; doi:10.1002/ece3.6987)
Supplement: Supplementary file 4 — Fig S4 [file ECE3-10-13949-s004.pdf]

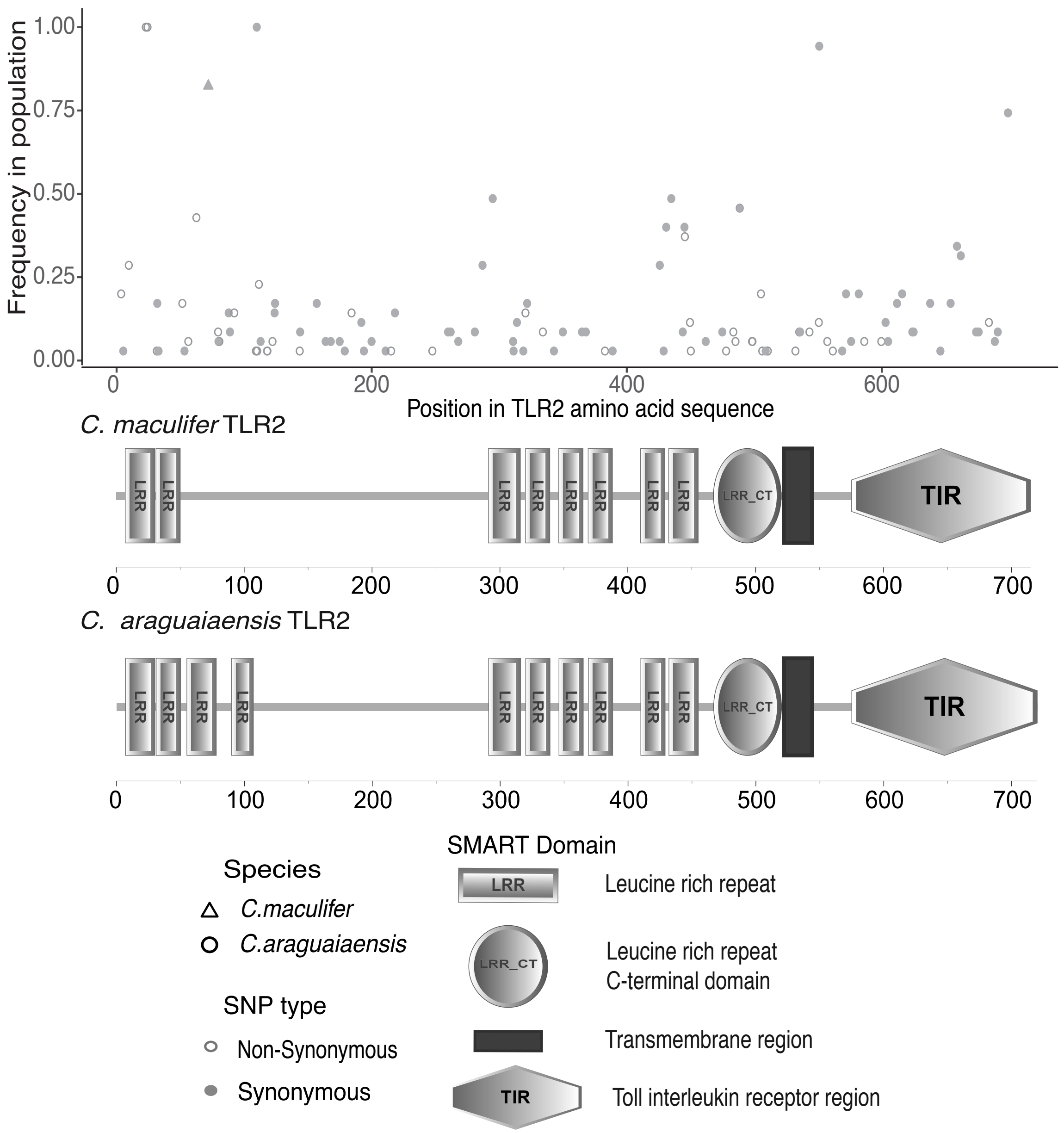

Frequency in population
1.00
0.75
0.50
0.25
0.00
0
200
400
600
Position in TLR2 amino acid sequence
C. maculifer TLR2
LRR
LRR_CT
TIR
0
100
200
300
400
500
600
700
C. araguaiaensis TLR2
SMART Domain
Species
C.maculifer
C.araguaiaensis
SNP type
Non-Synonymous
Synonymous
Leucine rich repeat
Leucine rich repeat C-terminal domain
Transmembrane region
Toll interleukin receptor region
