# Supplementary material for: Help or hindrance? The evolutionary impact of whole‐genome duplication on immunogenetic diversity and parasite load
Source: Ecol Evol. 2020 Nov 22;10(24):13949–56. doi: 10.1002/ece3.6987 (PMC7771170; doi:10.1002/ece3.6987)
Supplement: Supplementary file 5 — Table S1 [file ECE3-10-13949-s005.docx]

Supplementary material

Table S 1. PCR forward and reverse primers for 2.5kb of the expressed region of TLR1 and TLR2 for *Corydoras* lineages 2-9 (univ) and specific for *C. maculifer* (Mac).

| **Primer name** | **Forward** | **Reverse** | **Annealing temperature (^o^C)** |
| --- | --- | --- | --- |
| TLR1_univ | TGGCGATCCTGGTGGCCA | CTCTGCTTGGAGTGCTGCT | 69.8 |
| TLR2_univ | GCCAGCAGGATCTAAGCGAC | TCGTCCCTTTTTAGAGCGGCC | 69.8 |
| Mac_TLR1* | AGGATTCACTGGCTATTCTGGAGG | GCAATGGGGTTTGGTAAATCTCG | 63.0 |
| Mac_TLR2* | GACATTGAGATCATTAGCCAGCAG | CGGCTCTCAGATTGTTCCAGAA | 68.2 |

**C. maculifer* specific

Supplementary Figure S1: Proportion of host individuals harbouring parasitic infections per host species (*Corydoras maculifer* and *C. araguaiaensis*). Parasites categorised according to the host tissue type that they were found in. Fishers Exact Test (*p <0.0003).

Supplementary Figure S2: Number of parasites per infected host across the two species (*Corydoras maculifer and C. araguaiaensis*). Parasites categorised according to the host tissue type that they were found in. Medians are depicted by the central line per box and means are represented by diamonds. No significant differences were detected.

Supplementary Figure S3: Frequencies of alternative bases in TLR1 across populations of *Corydoras maculifer* and *C. araguaiaensis*, aligned to the protein domain output derived by SMART analysis for TLR1 for each species.

Supplementary Figure S4: Frequencies of alternative bases in TLR2 across populations of *Corydoras maculifer* and *C. araguaiaensis*, aligned to the protein domain output derived by SMART analysis for TLR1 for each species.
